# *Momordica balsamina* improves glucose handling in a diet-induced prediabetic rat model

**DOI:** 10.1371/journal.pone.0295498

**Published:** 2023-12-14

**Authors:** Bongiwe Khumalo, Angezwa Siboto, Akinjide Moses Akinnuga, Ntethelelo Sibiya, Andile Khathi, Phikelelani Siphosethu Ngubane

**Affiliations:** 1 Department of Physiology, School of Laboratory Medicine and Medical Science, College of Health Sciences, University of KwaZulu Natal, Durban, South Africa; 2 Department of Physiology, Faculty of Basic Medical Sciences, Cross River University of Technology, Okuku Campus, Cross River, Nigeria; 3 Pharmacology Division, Faculty of Pharmacy, Rhodes University, Grahamstown, South Africa; Tongji University Tenth People’s Hospital: Shanghai Tenth People’s Hospital, CHINA

## Abstract

Prolonged exposure to high energy diets has been implicated in the development of pre-diabetes, a long-lasting condition that precedes type 2 diabetes mellitus (T2DM). A combination of pharmacological treatment and dietary interventions are recommended to prevent the progression of pre-diabetes to T2DM. However, poor patient compliance leads to negligence of the dietary intervention and thus reduced drug efficiency. *Momordica balsamina* (MB) has been reported to possess anti-diabetic effects in type 1 diabetic rats. However, the effects of this medicinal plant in conjunction with dietary intervention on pre-diabetes have not yet been established. Consequently, this study sought to evaluate the effects of MB on glucose homeostasis in a diet-induced pre-diabetes rat model in the presence and absence of dietary intervention. Pre-diabetes was induced on male Sprague Dawley rats by a high fat high carbohydrate (HFHC) diet for a period of 20 weeks. Pre-diabetic male Sprague Dawley rats were treated with MB (250 mg/kg p.o.) in both the presence and absence of dietary intervention once a day every third day for a period of 12 weeks. The administration of MB with and without dietary intervention resulted in significantly improved glucose homeostasis through reduced caloric intake, body weights, with reduced plasma ghrelin concentration and glycated hemoglobin by comparison to the pre-diabetic control. MB administration also improved insulin sensitivity as evidenced by the expression of glucose transporter 4 (GLUT 4) and glycogen synthase on the prediabetic treated animals. These results suggest that MB has the potential to be used to manage pre-diabetes and prevent the progression to overt type 2 diabetes as it demonstrated the ability to restore glucose homeostasis even in the absence of dietary and lifestyle intervention.

## Introduction

Prediabetes is a prominent risk factor for the onset of type 2 diabetes [1]. Prediabetes is a metabolic complication that is characterized by impaired fasting glucose (IFG), impaired glucose tolerance (IGT) and elevated glycated hemoglobin (HbA1c) [2]. Literatures have shown that impaired glucose homeostasis is a result of resistance of insulin receptor substrate 1 (IRS-1) to insulin in targeted tissues [3]. Under physiological conditions, tissues such as the skeletal muscle, liver and adipose play a crucial role in maintaining blood glucose [4]. However, during insulin resistance, the insulin sensitivity of these tissues is decreased, thus, resulting into a slightly elevated fasting blood glucose concentration [5]. The slightly elevated blood glucose or moderate hyperglycaemia causes metabolic complications such as dysregulation of the hunger hormone (ghrelin), ectopic lipid deposition and glycation of proteins such as hemoglobin [6].

Consequently, the epidemiological findings on prediabetes are a great concern. Researchers have estimated that the global prevalence of prediabetes is expected to rise above 470 million by 2030 [7]. The rise in the incidence of T2DM over recent years, and its ever earlier age at presentation tends to have dominated the field, yet T1DM has behaved in an exactly parallel manner [8]. Literature have also shown that unhealthy eating habits which include high carbohydrates and high fats diet are responsible for the high incidence of prediabetes [9, 10]. Chronic ingestion of a high-fat high-carbohydrate diet has been shown to compromise the sensitivity of peripheral tissues to insulin, resulting in hyperglycaemia, prediabetes or diabetes [11]. However, a change of diet to a lower-caloric diet has been reported to reduce blood glucose and increase the sensitivity of these tissues to glucose uptake [12]. Despite a change in diet recommendation, some individuals cannot strictly adhere to the diet regimen, thus posing a challenge in the management of prediabetes with only western medicine without diet intervention [13]. Therefore, there is a need for alternative treatment strategies that would be effective in both the presence and absence of dietary intervention with minimal undesirable side effects.

Literature has reported that medicinal plant extracts ameliorate glucose impairment in streptozotocin (STZ) diabetic rats [14]. Evidently from our laboratory, medicinal plant extracts such as *Syzygium cordatum* and *Syzygium aromaticum*, have been shown to exert hypoglycaemic effects by stimulation of glucose uptake via increased translocation of GLUT 4 transporters in a streptozotocin (STZ)-induced type 1 diabetic rat model [15–17]. The effects of MB in insulin resistant diabetic models have not been shown. In addition, the effects of MB to improve hyperglycaemic conditions in the absence of diet intervention have not been shown. Therefore, in this study, we sought to investigate the effects of *Momordica balsamina* on glucose handling in a diet-induced prediabetic rat model. This will be both in the presence and absence of diet intervention.

## Methodology

### Plant extraction

*Momordica balsamina* (MB) leaves were used and were harvested at the University of KwaZulu-Natal (UKZN), Westville campus premises and identified by Baijnath, a botanist, at UKZN, Westville campus, Durban, South Africa. Thereafter, the leaves were rinsed three times with water to remove any residual dirt.

Methanolic extract of MB leaves was prepared via a standard protocol that has been validated in our laboratory [18]. Briefly, the air-dried MB leaves were sequentially extracted twice at 24 hours intervals by using methanol (45 mL) and deionized water (45 mL) at room temperature. The solvent was removed from the crude extract under reduced pressure at 55±1ºC through a rotatory evaporator to yield dichloromethane (DCMS) and ethyl acetate (EAS) soluble.

### Cytotoxicity studies

Cell viability was conducted in C2C12 muscle cell lines to examine the cytotoxic effects *of M*. *balsamina*. Cell viability of C2C12 muscle cell lines was measured by means of 3-(4,5-Dimethylthiazol-2-yl)-2,5-diphenyltetrazolium bromide (MTT) assay originally described by Mosmann (1983). Cells were trypsinised and seeded into 96-well plates (Bibby-Sterilin, Staffordshire, England) at a seeding density of 1.8 x 104 cells/well and incubated for 24 h to permit attachment and growth of cells to semi-confluency. Thereafter, the medium (0.5 ml) was replaced and three doses of *M*. *balsamina* (50, 25 and 12.5 μmol/L) were added to the wells and incubated at 37ºC for 12, 24 and 48 h respectively. After each incubation period, the medium was removed and MTT solution (5 mg/ml in phosphate buffered saline, 20 μL) was and media (100 μL) were added to each well. The cells were incubated for 4 h to allow for the formation of blue formazan crystals. After 4 h incubation DMSO (100 μl/well) was added and incubated at 37ºC for an hour into each well. The absorbance was measured at 570 nm in a UV-visible spectrophotometer (Thermoscientific Biomate, Cambridge, UK).

The percentage cell viability was calculated as follows: Cell viability (%) = [A570 treated cells − background] / [A570 control cells − background] x 100.

### Animal studies

Thirty-six (36) male Sprague-Dawley rats (150-180g) were used in this study. The animals were bred in the Biomedical Resource Unit (BRU) of the UKZN. The animals were maintained under standard environmental conditions of constant temperature (22±2°C), CO_2_ content (<5000 p.m.), relative humidity (55±5%) and illumination (12 h light/dark cycle). The animals were allowed access to standard rat chow (Meadow Feeds, South Africa) and water *ad libitum*, and acclimatized for 2 weeks before exposure to an established experimental high-fat high-carbohydrate (HFHC) diet which is supplemented with 15% fructose drinking water. The HFHC diet was formulated to consist of carbohydrates (55% Kcal/g), fats (30% Kcal/g), and proteins (15% Kcal/g) as described in previous study [19].

### Induction of prediabetes

Thirty (30) experimental animals were exposed to HFHC diet and 15% fructose drinking water for 20 weeks to induce prediabetes while six (6) normal control rats were exposed to standard chow for the equal number of weeks as previously described [20]. After 20 weeks, the animals that exhibited impaired fasting blood glucose concentrations (5.6–6.9 mmol/L) and/or impaired glucose tolerance (7.0–11.0 mmol/L) were considered prediabetic according to American Diabetes Association (ADA) criteria.

### Experimental design

The study included two major groups, the normal control group and pre-diabetic group. The pre-diabetic group was further sub-divided into six groups (n = 6 in each group). The groups were categorized as follows: the pre-diabetic control (PC), which continued with the experimental diet throughout the study period; metformin group (Met + HFHC) which are the pre-diabetic animals that continued with the experimental diet but received metformin during treatment period; metformin and diet intervention group (Met + DI) which are the pre-diabetic animals that changed to a normal diet and received metformin during treatment period; *Momordica balsamina* group (MB + HFHC) which are the pre-diabetic animals that continued with experimental diet but received MB during treatment period as well as MB and diet intervention group (MB + DI) which are the pre-diabetic animals that changed to a normal diet and received oleanolic acid during experimental period. The normal control group was the group of animals that were fed normal diet and diagnosed as without pre-diabetes.

### Treatment of animals

After prediabetic induction, the animals were divided into 6 groups (n = 6) and treated for 12 weeks. The animals were treated orally once every third day at 9h00 am. The Group 1 (NC) rats received as vehicle 3ml/kg of diluted dimethyl sulphoxide, DMSO (2ml DMSO:19ml normal saline) and continuously fed on normal diet (ND). Group 2 (HFHC) rats were untreated prediabetic rats that continuously fed on HFHC diet and received 3ml/kg of diluted DMSO. Group 3 (ND + MB) rats changed diet to ND (i.e. diet intervention) and received MB (250 mg/kg p.o). Group 4 (HFHC + MB) animals continuously fed on HFHC diet (i.e. without diet intervention) and received MB (250 mg/kg p.o.). Group 5 (ND + MB) animals changed diet to ND and received metformin (MET) (500 mg/kg p.o) while Group 6 (HFHC + MET) rats continuously fed on HFHC diet and received metformin (500 mg/kg p.o.). In each group, measured parameters such as body weight, food and fluid intake, urinary output were measured by placing the animals in metabolic cages for 24 h (Tecniplast; Labotec, Cape Town, South Africa), while blood glucose concentration was determined by using a One-Touch select glucometer (Lifescan, Malta, United Kingdom) via the tail-prick method. The treatment period lasted for 12 weeks. The doses administered to rats for metformin and MB in the present study were extrapolated from previous studies and tested by MTT assay [20, 21].

### Caloric intake

At every 4^th^ week of the treatment period, the caloric intake of all the animals was determined via food and water intakes measurement by placing the animals in the metabolic cages (Tecniplast; Labotec, Cape Town, South Africa).

### Blood glucose concentration

At every 4^th^ week of treatment, the fasting blood glucose concentration (FBG) was determined by using One-Touch select glucometer (Lifescan, Malta, United Kingdom) via the tail-prick method.

### Oral glucose tolerance test (OGTT)

In the 12^th^ week of treatment, the OGTT was conducted after glucose loading. The OGTT responses were monitored in all the animal groups through previously described protocol [20]. In brief, after a 12-hour fasting period, FBG was measured (time, 0 min) in all the animals. Thereafter, the animals were loaded with glucose (0.86 g/kg, p.o.) via oral gavage. The glucose concentrations were measured at 15,30,60, and 120 min following glucose loading using a One-Touch select glucometer (Lifescan, Malta, United Kingdom) via the tail-prick method.

### Blood collection and tissue harvesting

All animals were anaesthetized with Isofor (100 mg/kg) (Safe line Pharmaceuticals (Pty) Ltd, Roodepoort, South Africa) in a gas anaesthetic chamber (Biomedical Resource Unit, UKZN, Durban, South Africa) for 3 minutes. While the rats remained unconscious, blood samples were collected by cardiac puncture and then injected into individual pre-cooled heparinised containers. The collected blood samples were centrifuged (Eppendorf centrifuge 5403, Germany) at 4°C, 503 g for 15 minutes to obtain plasma. Thereafter, the plasma samples were stored at -80°C in a Bio Ultra freezer (Snijers Scientific, Netherland) until ready for biochemical analysis. Also, the liver and skeletal muscle (gastrocnemius) tissues were removed from the animal, rinsed, weighed and snap frozen in liquid nitrogen before storage at -80°C in a Bio Ultra freezer for biochemical analysis.

### Biochemical analysis

Glycated hemoglobin (HbA1c) and ghrelin concentrations were measured using their respective ELISA kits (Elab science Biotechnology Co., Ltd., Houston, TX, USA) according to the manufacturer’s instructions. Plasma insulin concentration was also measured by using an ultrasensitive rat insulin ELISA kit (Mercodia AB, Sylveniusgatan 8A, SE-754 50, Uppsala, Sweden) according to the manufacturer’s instructions. Homeostasis model assessment (HOMA2-IR) index in each animal was determined from the product of fasting insulin concentration (FIC) and fasting blood glucose (FBG) divided by 22.5 i.e.

HOMA2-IR=FIC×FBG/22.5

### Glycogen assay

Glycogen analysis was performed in the muscle and liver tissues by using a previously described laboratory protocol [20]. The assay absorbance was measured at 620 nm by using the Spectrostar Nano spectrophotometer (BMG Labtech, Ortenburg, LGBW Germany). The glycogen concentrations were calculated from the glycogen standard curve. The standard curve ranges from 200 to 1000 mg/L.

### Western blot analysis

The harvested skeletal muscle tissues of animals in each group were analysed for GLUT 4 and glycogen synthase via western blotting. The tissues (0.1 g) were homogenized on ice in isolation buffer (0.5 mM Na_2_EDTA, 0.1 M KH_2_PO_4_, 0.1 mM dithiothreitol, 0.25 M sucrose) and then centrifuged at 400 x g for 10 min (4°C). The protein content was quantified by using the Lowry method. All the samples were standardized to one concentration (1 mg/ml). The proteins were then denatured by boiling in laemmli sample buffer (0.5 M Tris-HCl, glycerol, 10% sodium dodecyl sulphate (SDS), 2-mercaptoethanol, 1% bromophenol blue) for 5 mins. The denatured proteins were loaded (25 μl) on prepared resolving (10%) and stacking (4%) polyacrylamide gels along with 5 μl of molecular weight marker. The gel was electrophoresed for 1 h at 150 V in electrode (running) buffer (Tris base, glycine, SDS, pH 8.3). Following electrophoresis, the resolved proteins were electro-transferred to an equilibrated polyvinylidene difluoride (PVDF)/ membrane for 1 h in transfer buffer (192 mM glycine, 25 mM Tris, 10% methanol). After transfer, the membrane was blocked with 5% non-fat dry milk in Tris-buffered saline with 0.1% Tween 20 (TTBS) (20 mM Tris, 150 mM NaCl, KCL, 0.05% Tween-20). The membranes were then immuno-probed with antibodies of GLUT 4 or glycogen synthase (1:1000 in 1% BSA, Neogen, USA) for 1 h at room temperature (RT). The PVDF membrane was then subjected to 5 washes (10 min each with gentle agitation) with TTBS. The membranes were then incubated in horse radish peroxidase (HRP)-conjugated secondary antibody (rabbit anti-mouse 1:10 000; Bio-Rad) for 1 h at RT. After further washing, antigen-antibody complexes were detected by chemiluminescence using the Immune-star^™^ HRP substrate kit (Bio-Rad, Johannesburg, South Africa). Chemiluminescent signals were detected with the Chemi-doc XRS gel documentation system and analysed using the quantity one software (Bio-Rad, Johannesburg, South Africa). Band intensity analysis was conducted on the resultant bands.

### Statistical analysis

All data were expressed as mean ± SEM. The statistical data comparisons were analysed via Graph pad Software version 7 by using one-way analysis of variance (ANOVA) with Tukey as post hoc test. A value of p < 0.05 was considered statistically significant.

### Ethical considerations

Animal Research Ethics Committee (AREC) of UKZN approved all experimental procedures with ethics number, AREC/062/018M.

## Results

### Cell viability

#### Effects of MB on cell viability

**Fig 1:**
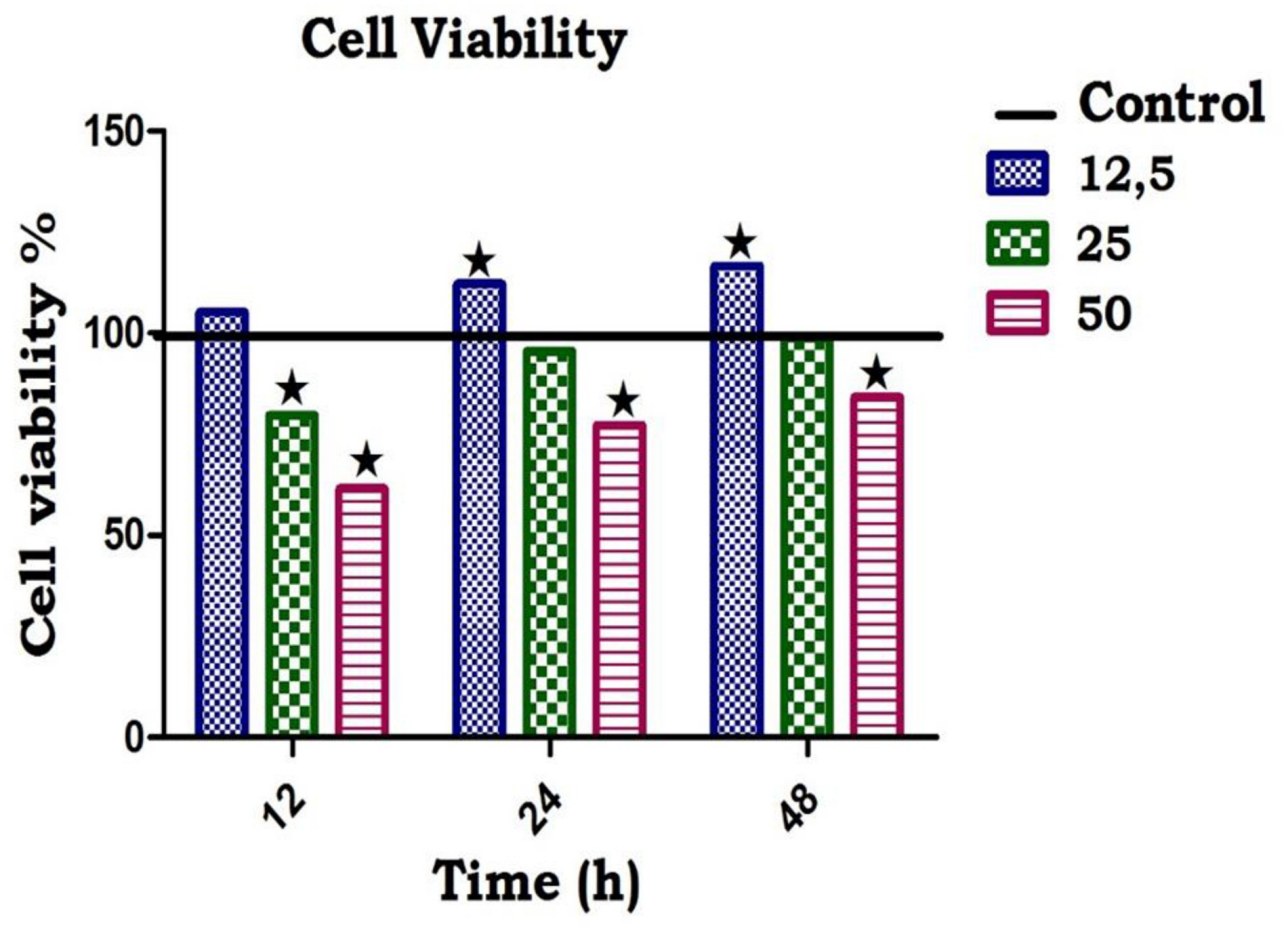
The effects of *Momordica balsamina* on cell viability on C2C12 muscle cell after 48 -hours of treatment period.

Fig 1 shows the effects of *M*. *balsamina* on cell viability of C2C12 skeletal muscle cell line using MTT assay. By comparison with the control group, the administration of 3 doses of *M*. *balsamina* (12.5, 25, and 50 mmol/L) showed no toxic effects at corresponding time intervals over the 48h incubation period. The metformin treatment group showed no significant decline in cell viability over the 48h treatment period.

### Effects of MB on body weight

The untreated prediabetic group high fat high carbohydrate (HFHC) had a significantly increased body weight and increased changes in body weight in comparison to normal control rats over the 12 weeks experimental period (Table 1). However, the administration of MB with and without dietary intervention resulted into a significant decrease in body weight and percentage changes in body weight when compared to the untreated prediabetic rats (HFHC) (p < 0.05) as indicated in Table 1. On the other hand, administration of metformin (Met) resulted into a significantly steady growth in week 0 and week 8, however, in week 12, a significant decrease in body weight was observed in comparison to HFHC group. (p < 0.05) Table 1.

**Table 1:** Effects of MB on body weight and percentage changes in body weight of prediabetic rats that changed to normal diet (ND) and those that continuously fed on HFHC diet for 12 weeks treatment period. Values are presented as mean ± SEM (n = 6) in each group.

Experimental groups	Week 0	Week 4	Week 8	Week 12
**NC**	400.80 ± 7.61 100%	415.80 ± 14.70 3.64%	420.20 ± 12.82 4.84%	427.80 ± 17.19 6.74%
**HFHC**	416.00 ± 24.41 100%	498.20 ± 18.91^α^ 19.76%^α^	526.20 ± 8.93^α^ 26.49%^α^	536.60 ± 15.43^α^ 28.99%^α^
**ND + MB**	411.20 ± 18.41 100%	418.00 ± 16.33* 1.65%*^α^	415.20 ± 13.69* 0.97%*^α^	428.60 ± 14.00* 4.23%*
**HFHC + MB**	409.80 ± 10.71 100%	472.00 ± 17.64^α^ 15.18%^α^	457.60 ± 14.11* 11.66%*^α^	468.60 ± 20.01* 14.35%*^α^
**ND + Met**	426.80 ± 17.23 100%	469.00 ± 16.10*^α^ 9.89%*^α^	478.40 ± 16.11*^α^ 12.09%*^α^	476.00 ± 18.96*^α^ 11.52%*^α^
**HFHC + Met**	400.40 ± 8.97 100%	482.40 ± 20.37^α^ 20.48%*^α^	499.40 ± 14.40^α^ 24.73%*^α^	488.00 ± 14.88*^α^ 21.88%*^α^

Values are presented as mean ± SEM

^α^p < 0.05 denotes comparison with normal control (NC).

*p < 0.05 denotes comparison with untreated prediabetic (HFHC).

Normal Control = NC, High fat high carbohydrate = HFHC, Normal diet + *Momordica balsamina* = ND + MB, High fat high carbohydrate + *Momordica balsamina* = HFHC + MB, Normal Diet + Metformin = ND + Met, High fat high carbohydrate + Metformin = HFHC + Met.

### Effects of MB on caloric intake

As shown in Table 2, there was a significant increase in the caloric intake of untreated prediabetic rats especially at 8^th^ and 12^th^ week when compared to the NC rats, and MB or metformin treated rats with or without dietary intervention.

**Table 2:** Effects of MB on caloric intake (kcal/g) of prediabetic rats that changed to normal diet (ND) and those that continuously fed on HFHC diet.

Experimental groups	Week 0	Week 4	Week 8	Week 12
**NC**	109±1.90 (100%)	125.04±2.40 ↑ (14.52%)	165.04±1.61 ↑ (51.16%)	178.40±0.87 ↑ (63.34%)
**HFHC**	121.47±1.01 (100%)	140.90±0.64* ↑ (15.99%)	206.58±0.84* ↑ (70.07%)	230.01±0.85* ↑ (89.36%)
**ND + MB**	119.58±0.51 (100%)	119.04±0.86*^α^ ↓ (0.40%)	157.59±2.80*^α^ ↑ (25.52%)	168.85±2.23^α^ ↑ (43.58%)
**HFHC + MB**	141.35±0.03 (100%)	105.94±2.00*^α^ ↓ (20.26%)	166.09±1.73^α^ ↑ (19.76%)	184.26±1.99*^α^ ↑ (49.03%)
**ND + Met**	115.02±0.67 (100%)	102.69±1.17*^α^ ↓ (10.72%)	120.51±0.75*^α^ ↑ (4.77%)	144.72±1.64*^α^ ↑ (25.82%)
**HFHC + Met**	118.09±0.51 (100%)	100.54±0.98*^α^ ↓ (14.86%)	99.51±1.52*^α^ ↓ (15.73%)	151.66±0.69*^α^ ↑ (25.82%)

Values are presented as mean ± SEM.

^α^p < 0.05 denotes comparison with normal control (NC).

*p < 0.05 denotes comparison with untreated prediabetic (HFHC).

(↑ = increase and ↓ = decrease). Normal Control = NC, High fat high carbohydrate = HFHC, Normal diet + *Momordica balsamina* = ND + MB, High fat high carbohydrate + *Momordica balsamina* = HFHC + MB, Normal Diet + Metformin = ND + Met, High fat high carbohydrate + Metformin = HFHC + Met.

### Effects of MB on fasting blood glucose, insulin concentration and HOMA2-IR index

The results showed that the HFHC group had a significantly increased fasting blood glucose, fasting insulin concentration and HOMA2-IR index in comparison to normal control (NC) group (Table 3). The MB treated prediabetic rats with or without diet intervention had a significant decrease in same parameters when compared to HFHC (p < 0.05) as shown in Table 3. Similarly, metformin treatment showed similar effects in both groups that underwent diet intervention and those that continued with the HFHC diet.

**Table 3:** The effects of MB on fasting blood glucose, insulin concentration and HOMA2-IR index in prediabetic rats that changed to normal diet (ND) and those that continuously fed on HFHC diet.

Groups	Fasting blood glucose (mmol/L)	Fasting blood insulin (mU/L)	HOMA2-IR index value
NC	4.30±0.51	7.84±0.38	0.95±0.16
HFHC	5.68±0.44^α^	9.66±0.55^α^	3.95±0.66^α^
ND + MB	4.80±0.29*	5.89±0.64^α^*	1.00±0.11*
HCHF +MB	4.65±0.72*	4.68±3.08^α^*	1.05±0.09*
ND + Met	5.28±0.14^α^	7.84±0.74*	1.00±0.09*
HFHC + Met	4.85±0.25*	10.52±1.37^α^*	1.05±0.09*

Values are presented as mean ± SEM.

^α^p < 0.05 denotes comparison with normal control (NC).

*p < 0.05 denotes comparison with untreated prediabetic (HFHC).

Normal Control = NC, High fat high carbohydrate = HFHC, Normal diet + *Momordica balsamina* = ND + MB, High fat high carbohydrate + *Momordica balsamina* = HFHC + MB, Normal Diet + Metformin = ND + Met, High fat high carbohydrate + Metformin = HFHC + Met.

### Effects on glycated hemoglobin (HbA1c) and oral glucose tolerance test (OGTT)

**Fig 2:**
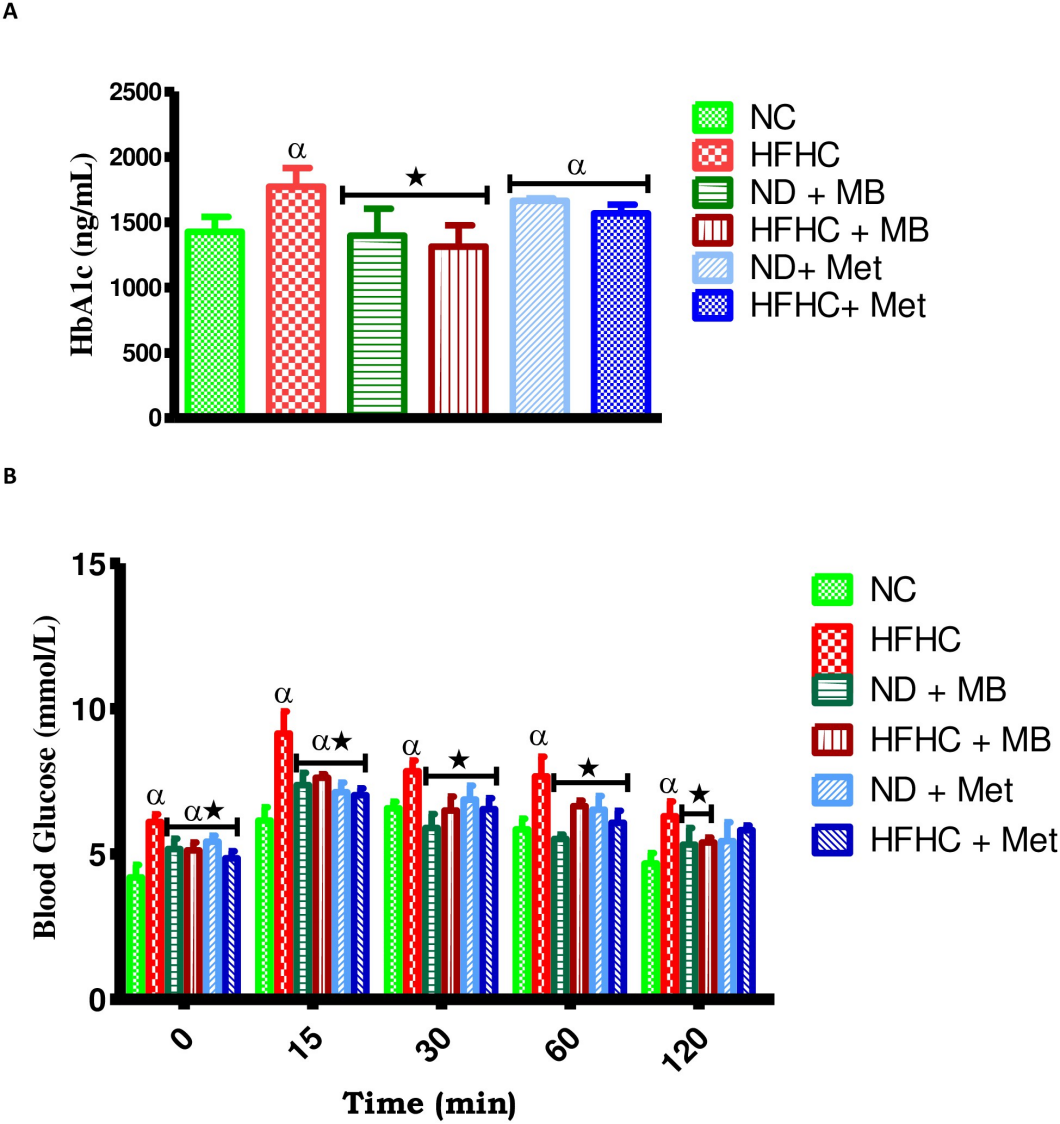
Effects of MB on (A) glycated hemoglobin (HbA1c) and (B) oral glucose tolerance test (OGTT) of prediabetic rats that changed to normal diet (ND) and those that continuously fed on HFHC diet. Values are presented as mean ± SEM. ^α^p < 0.05 denotes comparison with normal control (NC). *p < 0.05 denotes comparison with untreated prediabetic (HFHC). Normal Control = NC, High fat high carbohydrate = HFHC, Normal diet + *Momordica balsamina* = ND + MB, High fat high carbohydrate + *Momordica balsamina* = HFHC + MB, Normal Diet + Metformin = ND + Met, High fat high carbohydrate + Metformin = HFHC + Met.

As indicated in Fig 2A, the results showed that the HbA1c concentration of untreated HFHC group was significantly higher in comparison to normal control (NC) group. Conversely, the administration of MB with and without diet intervention resulted in a significantly decreased HbA1c concentration when compared to HFHC (Fig 2A).

As demonstrated in Fig 2B, the results showed that the untreated prediabetic rats (HFHC) had a significantly increased blood glucose concentration throughout the time interval of the glucose tolerance response when compared to NC rats. However, administration of both MB and metformin significantly decreased the blood glucose concentration at 60 and 120 min with or without diet intervention in comparison to the HFHC group (Fig 2B).

### Effects of MB on ghrelin

**Fig 3:**
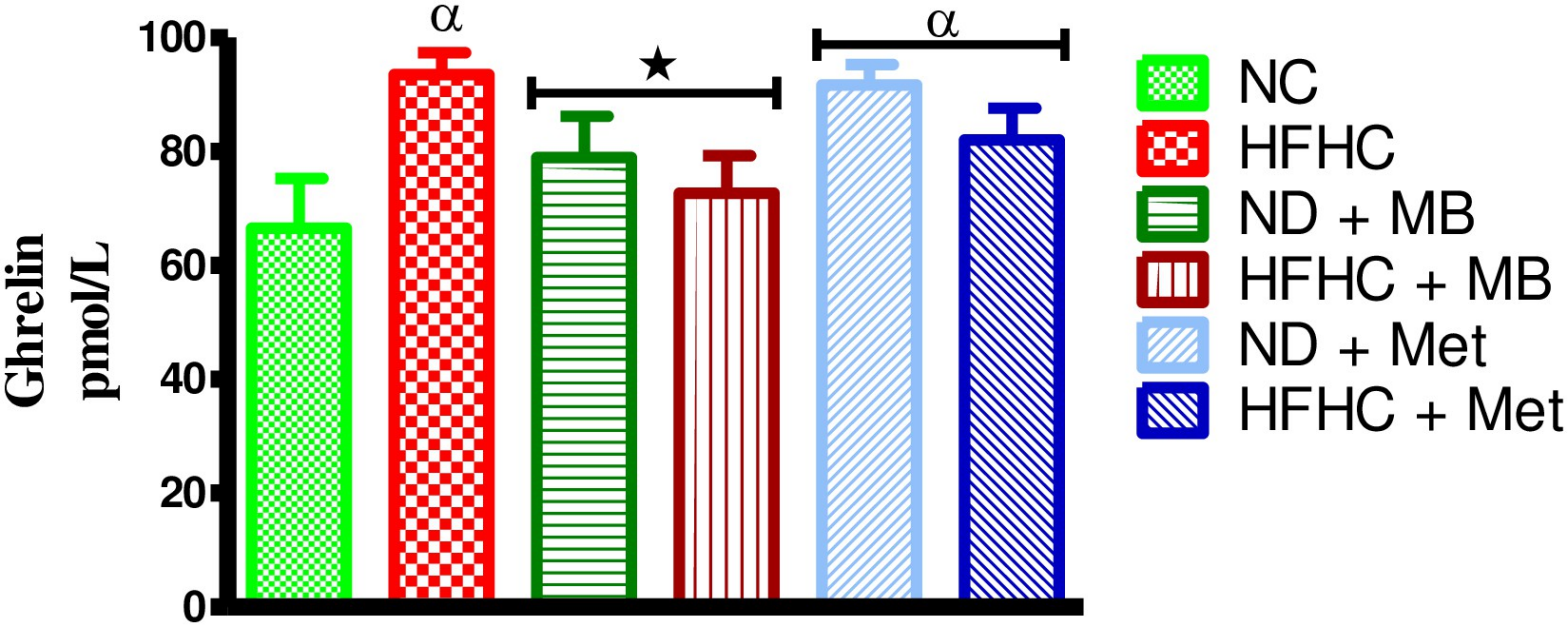
Effects of MB on plasma ghrelin concentrations of prediabetic rats that changed to normal diet (ND) and those that continuously fed on HFHC diet. Values are presented as mean ±SEM. ^α^p < 0.05 denotes comparison with normal control (NC). *p < 0.05 denotes comparison with untreated prediabetic (HFHC). Normal Control = NC, High fat high carbohydrate = HFHC, Normal diet + *Momordica balsamina* = ND + MB, High fat high carbohydrate + *Momordica balsamina* = HFHC + MB, Normal Diet + Metformin = ND + Met, High fat high carbohydrate + Metformin = HFHC + Met.

The results showed that the untreated prediabetic rats (HFHC) and metformin treated prediabetic with or without diet intervention had a significantly increased plasma ghrelin concentration in comparison to NC rats as shown in Fig 3 (p < 0.05). However, the MB-treated prediabetic animals had a significantly decreased ghrelin concentration when compared to HFHC animals. Conversely, the ghrelin concentration of metformin treated prediabetic rats was insignificant in comparison to HFHC rats.

### Effects of MB on skeletal muscle and liver glycogen concentrations

**Fig 4:**
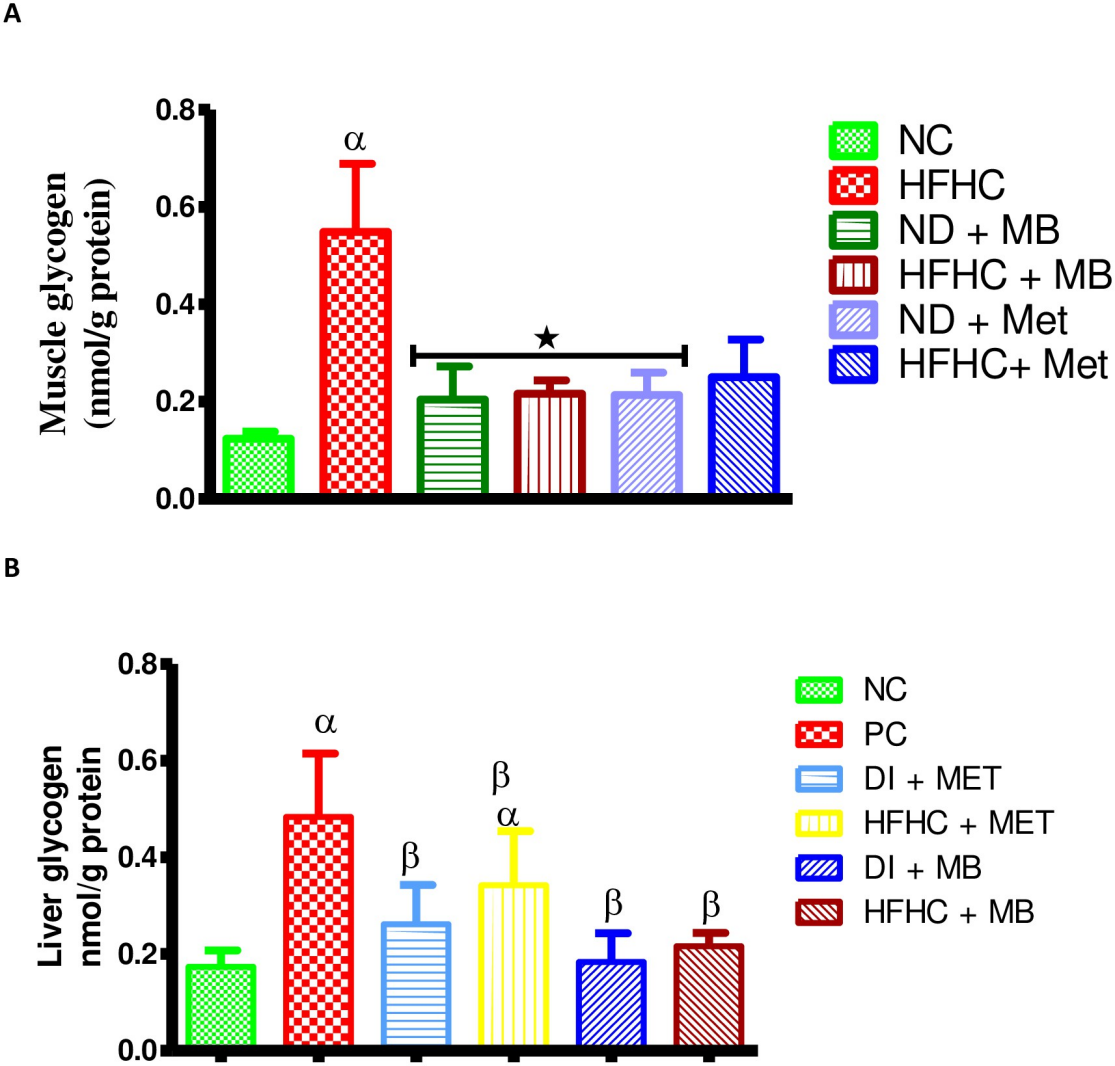
Effects of MB on (A) muscle glycogen concentration and (B) liver glycogen concentration of prediabetic rats that changed to normal diet (ND) and those that continuously fed on HFHC diet. Values are presented as mean ± SEM. ^α^p < 0.05 denotes comparison with normal control (NC). *p < 0.05 denotes comparison with untreated prediabetic (HFHC). Normal Control = NC, High fat high carbohydrate = HFHC, Normal diet + *Momordica balsamina* = ND + MB, High fat high carbohydrate + *Momordica balsamina* = HFHC + MB, Normal Diet + Metformin = ND + Met, High fat high carbohydrate + Metformin = HFHC + Met.

The results showed that HFHC group had a significantly increased skeletal muscle and liver glycogen concentrations in comparison to NC group, and MB or Met treated rats with diet intervention (Fig 4A and 4B). However, the liver glycogen concentration of the Met-treated prediabetic rats without diet intervention was significantly increased in comparison to NC rats while that of Met-treat prediabetic rats with diet intervention was insignificant as demonstrated in Fig 4B.

### Effects of MB on glucose transporter 4 (GLUT4) expression

**Fig 5:**
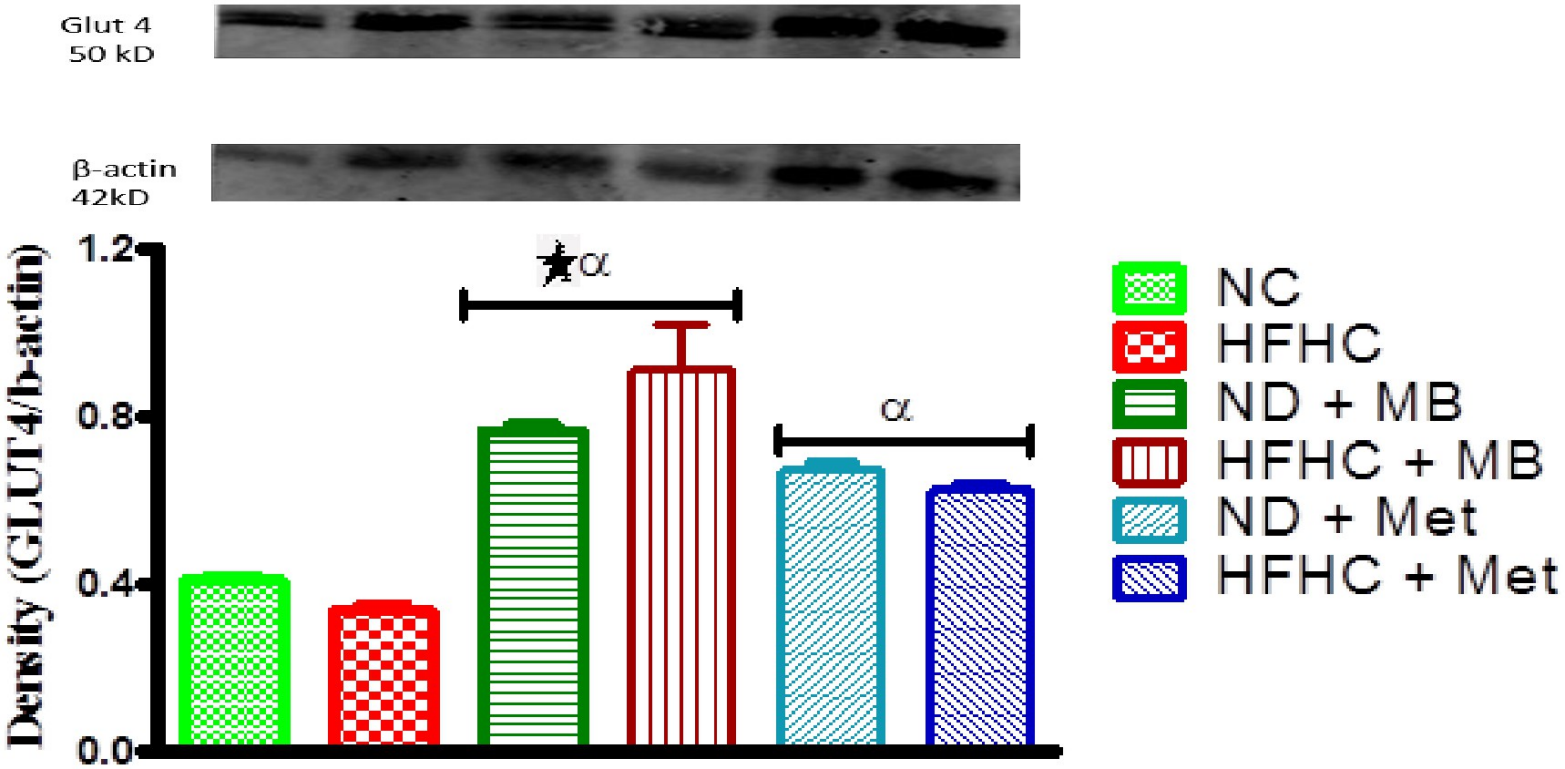
Effects of MB on GLUT 4 protein expression of prediabetic rats that changed to normal diet (ND) and those that continuously fed on HFHC diet. Values are presented as mean ± SEM. ^α^p < 0.05 denotes comparison with normal control (NC). *p < 0.05 denotes comparison with untreated prediabetic (HFHC). Normal Control = NC, High fat high carbohydrate = HFHC, Normal diet + *Momordica balsamina* = ND + MB, High fat high carbohydrate + *Momordica balsamina* = HFHC + MB, Normal Diet + Metformin = ND + Met, High fat high carbohydrate + Metformin = HFHC + Met.

The GLUT 4 protein expressions in the skeletal muscles of both MB-treated and Met-treated prediabetic rats with or without diet intervention were significantly increased in comparison to the untreated prediabetic (HFHC) rats as indicated in Fig 5. Also, there was a significant increase in the GLUT 4 protein expression of MB-treated prediabetic rats with or without diet intervention when compared to NC rats (p<0.05).

### Effects of MB on skeletal muscle and liver glycogen synthase expressions

**Fig 6:**
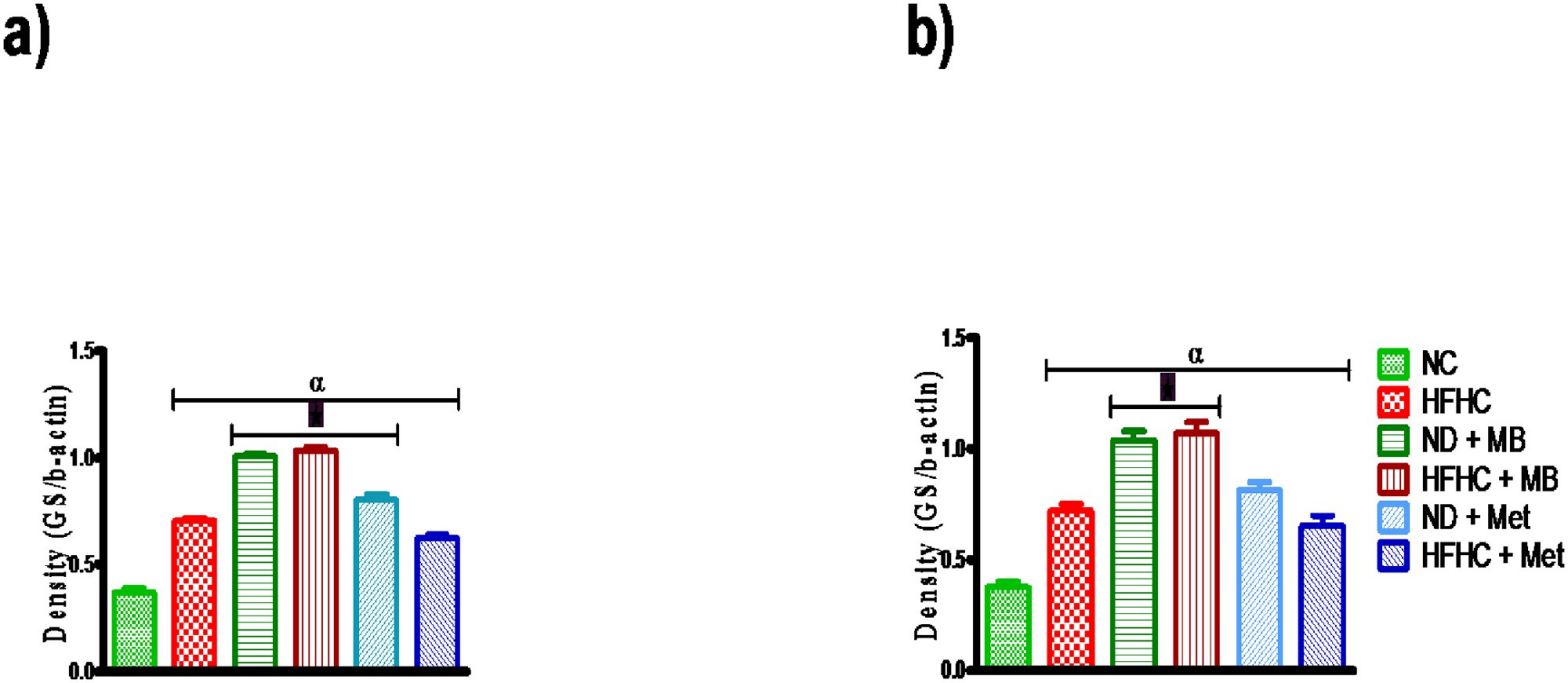
Effects of MB on (a) muscle glycogen synthase and (b) liver glycogen synthase expressions of prediabetic rats that changed to normal diet (ND) and those that continuously fed on HFHC diet. Values are presented as mean ± SEM. ^α^p < 0.05 denotes comparison with normal control (NC). *p < 0.05 denotes comparison with untreated prediabetic (HFHC). Normal Control = NC, High fat high carbohydrate = HFHC, Normal diet + *Momordica balsamina* = ND + MB, High fat high carbohydrate + *Momordica balsamina* = HFHC + MB, Normal Diet + Metformin = ND + Met, High fat high carbohydrate + Metformin = HFHC + Met.

As shown in Fig 6a, there was a significant increase in the expression of muscle glycogen synthase in both MB-treated prediabetic animals with or without diet intervention and Met-treated prediabetic animals with diet intervention in comparison to the untreated HFHC animals. Also, there was a significant increase in the glycogen synthase protein expression in the liver of MB-treated prediabetic animals with or without diet intervention in comparison to HFHC animals as indicated in Fig 6b. However, other experimental groups had a significant increase in the expression of glycogen synthase in both the muscle and liver when compared to the NC group (Fig 5a and 5b).

## Discussion

This study investigated the effects of *Momordica balsamina* (MB) on glucose handling in a high-fat high-carbohydrate diet-induced (HFHC) prediabetic rat model. Literature has reported that the leaves of the plant exhibits the maximum activity and the leaves contain important source of nutrients, having 17 amino acids with adequate mineral composition such as resins, alkaloids, flavonoids, glycosides, steroids, terpenes, cardiac glycoside. Many in vivo studies have demonstrated the relatively low toxicity of all parts of the bitter melon plant when ingested orally. The fruits and seeds of the plant has been demonstrated to possess toxicity [22, 23]. Many in vivo studies have demonstrated the relatively low toxicity of all parts of the bitter melon plant when ingested orally [22]. However, MB fruits and seeds have demonstrated to have toxicity [23]. Also, studies indicate that MB possesses hypoglycaemic, kidney dysfunction ameliorative effects and hepatoprotective properties in streptozotocin-induced diabetic rats [21, 24]. However, its glucose handling effects in high-fat high-carbohydrate induced prediabetes rat model have not been established.

Although studies have reported known toxic effects of fruits, previous studies have also reported the use of medicinal plants to be associated with toxicity when taken in high doses [25, 26]. In this study, we therefore used the MTT assay to investigate for cytotoxicity for the three doses of MB (12.5, 25 and 50 mmol) in C2C12 skeletal muscle cell line. All 3 doses of MB showed no cytotoxicity effects in C2C12 muscle cell lines.

According to American Diabetes Association (ADA), one of the diagnostic features for prediabetes is glycated hemoglobin (HbA1c) [27]. In this study, there was a significant increase in the concentration of HbA1c in untreated HFHC group, indicating the presence of hyperglycaemia and prediabetes. However, animals that were treated with MB and metformin showed a significant decrease in HbA1c compared to the untreated HFHC group. We therefore speculate that MB was able to decrease HbA1c through its known hypoglycaemic effect [28]. Several literatures have stated that HbA1c alone is not sufficient to diagnose diabetes as it has been shown to lack sensitivity [29–31]. Therefore, the HbA1c test is shown to be more effective when coupled with the oral glucose test (OGTT) [32]. On this basis, we performed an OGTT of which blood glucose remained high in untreated HFHC animals, suggesting that the animals were prediabetic since OGTT remained elevated after 2 hours of postprandial glucose feeding. MB treated groups however, showed a decrease in blood glucose levels in the 2 hours postprandial glucose feeding. This may be again due to the hypoglycaemic effects of MB [21]. Similarly, the animals that were treated with metformin showed a slight decrease in glucose levels. Metformin is known to activate the AMPK activity thereby inhibiting hepatic glucose production [33]. To further examine the mechanisms by which MB exerts its hypoglycaemic effects, we measured the energy regulating hormone, ghrelin, since it also plays a role in glucose homeostasis.

Ghrelin is a peptide that acts on the growth hormone (GH) secretagogue receptors in the pituitary and hypothalamus to stimulate food intake [34]. In diabetes, ghrelin has been shown to be secreted in excess, thus, leading to the development of polyphagia which was observed in this study [35]. Studies have also linked high ghrelin concentration with increased insulin secretion and development of obesity in diabetes which was also shown in untreated HFHC prediabetic rats in this study [20, 36]. The administration of both MB and metformin showed a decrease in ghrelin concentration, thus, decreasing caloric intake in these animals. Furthermore, MB and metformin maintained steady body weight gain in comparison to the untreated HFHC and normal control animals which showed an increase over the 12-week period. The regulation of caloric intake is essential in the maintenance of insulin sensitivity, body weights and hyperglycaemia in metabolic disorders [37]. Various medicinal plants have been shown to decrease ghrelin concentration through sensitizing the peripheral cells for insulin thereby suppressing ghrelin secretion, hence, decreasing caloric intake [20]. Despite the unclear mechanism by which MB reduces circulating ghrelin concentration, we postulate that MB may restore ghrelin regulation through sensitizing the peripheral cells for insulin and suppress ghrelin secretion. Interestingly, the hypoglycaemic effects of MB in the presence and absence of dietary intervention were proved by an improvement in parameters such as ghrelin, body weight and caloric intake. This is important since many conventional treatments have been rendered ineffective in the absence of dietary intervention due to poor patient compliance to dietary intervention [38, 39].

During insulin resistance, impairment of insulin receptor substrate-1(IRS-1) results in the inability of GLUT4 transporter to translocate to the surface of the membrane to facilitate glucose entry into the cell [40]. This was observed in the untreated HFHC diet group as there was a significant decrease in GLUT 4 protein expression. However, the administration of metformin and MB restored the GLUT 4 protein expression in both animals that underwent diet change and those that did not undergo diet intervention. This suggests that MB may increase glucose uptake by activating IRS-1 signaling, thus, increasing the expression of GLUT 4 transporters and reducing blood glucose levels independent to diet change [41].

Moreover, glycogen synthase (GS) is an enzyme that is responsible for the conversion of glucose to glycogen in the liver and skeletal muscle during fasting [42, 43]. Literature has shown that the overexpression and overactivity of glycogen synthase are associated with an impaired ability of insulin to activate glucose disposal [44]. Interestingly, in this study, HFHC diet fed animals demonstrated a slight increase in glycogen storage and an increase in glycogen synthase expression compared to normal control animals. Glycogen synthase kinase 3 (GSK-3) activity has been shown to be higher in insulin resistant tissues, thus causing impaired insulin action, and further resulting in high expression of glycogen synthase in diabetes [45]. Furthermore, this may be due to a high carbohydrate diet causing high levels of circulating free fatty acid which leads to an imbalance in the phosphorylation of glycogen synthase kinase [46]. Importantly, in this study, MB and metformin treatment increased glycogen concentration, protein expression of glycogen synthase and GLUT 4 expression with or without a change of diet compared to the untreated HFHC fed animals. In addition, a plant of the same genus *Momordica charantia* (MC) has been shown to achieve its homeostatic blood glucose by sensitizing the insulin receptor substrate 1(IRS-1) at the surface of the membrane leading to an increased GLUT 4 expression thereby promoting blood glucose uptake in animals [47, 48]. We therefore speculate that MB may also increase insulin sensitivity via IRS-1 since the protein expression of GLUT 4 was increased. Some studies reported that MC stimulates phosphoinositol-3-kinase which phosphorylates Akt and downregulates mTOR to attenuate insulin resistance [49, 50]. Indeed, this study showed that MB in both the presence and absence of diet intervention resulted in the reduction of insulin concentrations which was further evidenced by the decrease in HOMAR-IR index, hence, suggesting an attenuation in insulin resistance.

## Conclusions

In conclusion, this study reveals that medicinal plants such as *Momordica balsamina* can increase insulin sensitivity and improve glycaemic control in HFHC diet fed insulin-resistant prediabetic rats via increased protein expression of GLUT 4 and glycogen synthase. The results in this study suggest that MB improves insulin sensitivity in diet-induced pre-diabetes even in the absence of diet intervention. Therefore, MB can play a pivotal role in decreasing prediabetes incidence and its progression to overt type 2 diabetes without diet intervention. Ultimately, the observations support and encourage further evaluation of the *Momordica balsamina* as an alternative therapeutic remedy for pre-diabetes.

## Limitations and future studies

Limitations in this study include a shortened treatment period of 12 weeks. This was however due to ethical reasons. Future studies include investigating the effects of MB on adipose tissue and leptin concentration. This will be to further conclude on the mechanisms by which MB restores insulin sensitivity since leptin serves a primary role as an anti-obesity hormone also, is upregulated by insulin and cortisol and downregulated by catecholamines. Also, to further investigate the effects of MB in preventing mitochondrial dysfunction in cardiolipin content, citrate synthase, β-HAD, and ETC NADH oxidase activities to delay tissue damage /injury that maybe due to chronic oxidative stress production stimulated by hyperglycaemic conditions.

## Supporting information

S1 Fig (PDF)

S1 Raw data (DOCX)
