# Supplementary figures and images for: Momordica balsamina improves glucose handling in a diet-induced prediabetic rat model
Source: PLoS One. 2023 Dec 14;18(12):e0295498. doi: 10.1371/journal.pone.0295498 (PMC10721073; doi:10.1371/journal.pone.0295498)

2018-08-23 10hr 57min 38sec

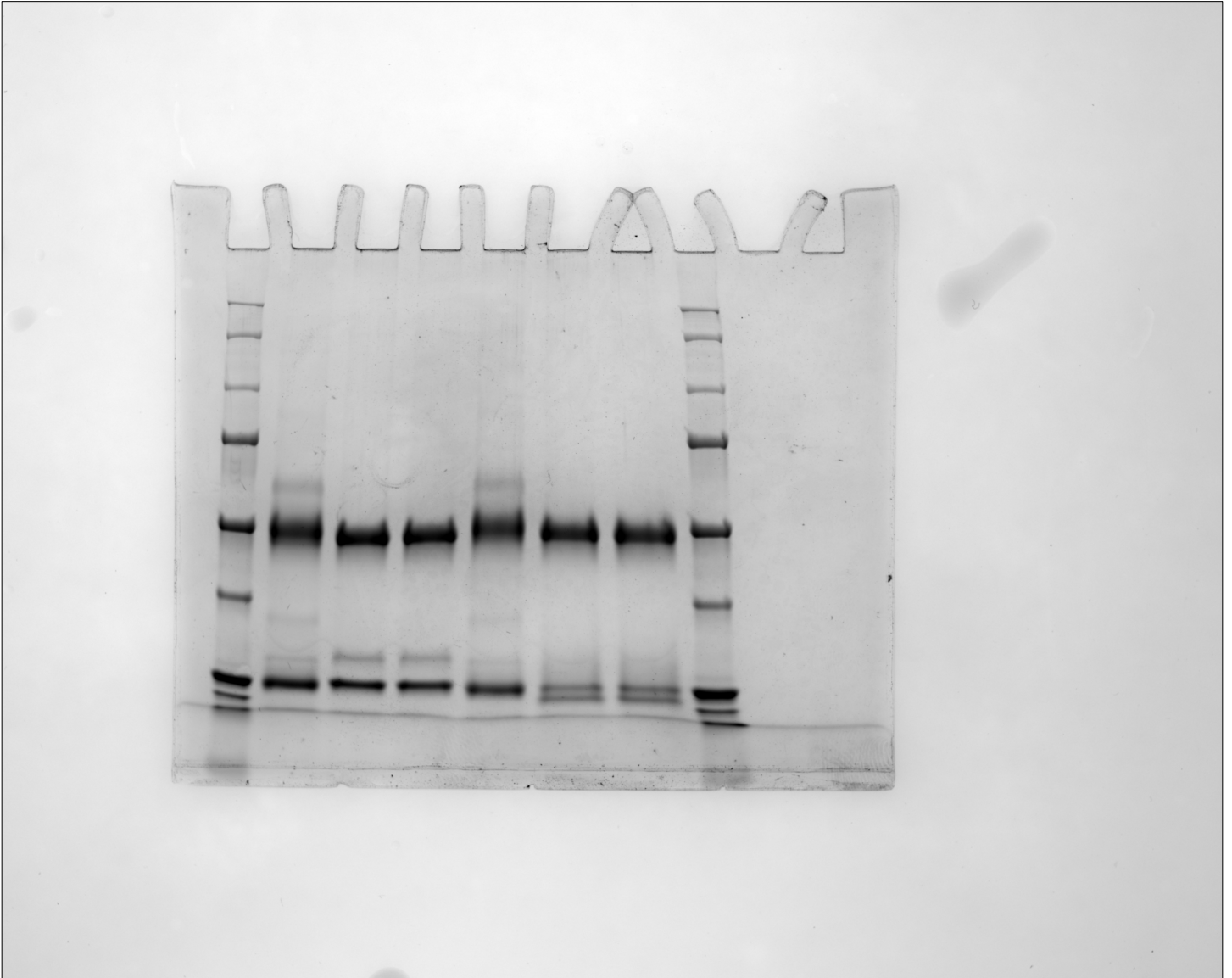

Supplement: S1 Fig — (PDF) [file pone.0295498.s001.pdf]
