# Supplementary material for: Momordica balsamina improves glucose handling in a diet-induced prediabetic rat model
Source: PLoS One. 2023 Dec 14;18(12):e0295498. doi: 10.1371/journal.pone.0295498 (PMC10721073; doi:10.1371/journal.pone.0295498)
Supplement: S1 Raw data — (DOCX) [file pone.0295498.s002.docx]

1.Glycated heam

| NC | PD | ND + MB | HFHC + MB | ND+ Met | HFHC+ Met |
| --- | --- | --- | --- | --- | --- |
| 1685.289 | 1875.837 | 1093.222 | 1107.050 | 1733.597 | 1502.793 |
| 1333.716 | 1679.922 | 1028.811 | 1148.536 | 1661.135 | 1464.520 |
| 1218.777 | 1194.160 | 2205.136 | 1181.786 | 1679.922 | 1605.358 |
| 1111.426 | 1650.400 | 1005.427 | 1044.213 | 1577.939 | 1351.683 |
| 1369.887 | 2181.786 | 1200.128 | 1297.307 | 1663.148 | 1822.161 |
| 1835.580 | 2052.965 | 1846.315 | 2105.734 | 1661.135 | 1655.768 |

OGGT

HFHC + MB HFHC + MB HFHC + MB HFHC + MB ND + Met ND + Met ND + Met ND + Met HFHC + MB HFHC + MB HFHC + MB HFHC + MB

4.9 4.6 5.9 5.1 5.8 5.7 4.9 5.3 5.4 5.1 4.3 4.6

7.7 7.3 7.6 7.9 7.5 6.2 7.7 7.1 6.8 6.6 7.0 7.7

5.6 7.7 6.8 5.9 7.9 5.6 7.1 6.9 7.4 6.7 5.6 6.5

6.6 6.7 7.1 6.2 5.2 7.4 6.6 6.9 7.0 5.0 6.2 6.1

5.2 5.3 5.2 5.9 3.7 6.8 5.6 5.7 5.6 6.3 5.7 5.7

Ghrelin

| NC | PD | ND + MB | HFHC + MB | ND+ Met | HFHC + Met |
| --- | --- | --- | --- | --- | --- |
| 87.785900 | 81.309740 | 100.018200 | 85.785900 | 95.749570 | 71.388540 |
| 99.045200 | 100.436200 | 99.330400 | 91.045200 | 100.463200 | 72.050440 |
| 50.304490 | 100.304500 | 60.600170 | 48.304490 | 81.045200 | 70.282730 |
| 51.680090 | 100.722900 | 60.965290 | 70.680090 | 100.145800 | 100.727800 |
| 50.256830 | 82.256830 | 71.071100 | 80.256830 | 82.050440 | 80.161500 |
| 60.034230 | 96.384460 | 81.547260 | 60.034230 | 90.934990 | 97.389700 |

Muscle glycogen

| NC | PD | ND + MB | HFHC + MB | ND+ Met | HFHC+ Met |
| --- | --- | --- | --- | --- | --- |
| 0.071000 | 0.952 | 0.080 | 0.2600 | 0.357 | 0.2960 |
| 0.168000 | 0.823 | 0.082 | 0.1990 | 0.257 | 0.1710 |
| 0.148000 | 0.294 | 0.422 | 0.2400 | 0.081 |  |
| 0.136000 | 0.337 | 0.304 | 0.3100 | 0.204 | 0.5330 |
| 0.098000 | 0.339 | 0.133 | 0.1550 | 0.171 | 0.1300 |
| 0.123000 |  |  | 0.1330 |  | 0.1230 |

Liver glycogen

| NC | PD | ND + MB | HFHC + MB | ND + Met | HFHC + Met |
| --- | --- | --- | --- | --- | --- |
| 0.0710 | 0.652 | 0.282 | 0.3600 | 0.357 | 0.4960 |
| 0.2680 | 0.823 | 0.280 | 0.3990 | 0.257 | 0.3710 |
| 0.2480 | 0.794 | 0.422 | 0.3400 | 0.381 | 0.8030 |
| 0.1360 | 0.837 | 0.304 | 0.3100 | 0.204 |  |
| 0.0980 | 0.839 | 0.233 | 0.2550 | 0.371 | 0.3300 |
| 0.2230 | 0.657 |  | 0.3330 | 0.601 | 0.3230 |
